# Multiparametric Analysis of Cell-Free DNA in Melanoma Patients

**DOI:** 10.1371/journal.pone.0049843

**Published:** 2012-11-27

**Authors:** Francesca Salvianti, Pamela Pinzani, Paolo Verderio, Chiara Maura Ciniselli, Daniela Massi, Vincenzo De Giorgi, Marta Grazzini, Mario Pazzagli, Claudio Orlando

**Affiliations:** 1 Department of Clinical Physiopathology, Clinical Biochemistry Unit, Università di Firenze, Firenze, Italy; 2 Unit of Medical Statistics and Biometry, Fondazione IRCCS, Istituto Nazionale dei Tumori, Milano, Italy; 3 Division of Pathological Anatomy, Department of Critical Care Medicine and Surgery, Università di Firenze, Firenze, Italy; 4 Department of Critical Care Medicine and Surgery, Dermatology Unit, Università di Firenze, Firenze, Italy; The Moffitt Cancer Center & Research Institute, United States of America

## Abstract

Cell-free DNA in blood (cfDNA) represents a promising biomarker for cancer diagnosis. Total cfDNA concentration showed a scarce discriminatory power between patients and controls. A higher specificity in cancer diagnosis can be achieved by detecting tumor specific alterations in cfDNA, such as DNA integrity, genetic and epigenetic modifications.

The aim of the present study was to identify a sequential multi-marker panel in cfDNA able to increase the predictive capability in the diagnosis of cutaneous melanoma in comparison with each single marker alone. To this purpose, we tested total cfDNA concentration, cfDNA integrity, *BRAF^V600E^* mutation and *RASSF1A* promoter methylation associated to cfDNA in a series of 76 melanoma patients and 63 healthy controls. The chosen biomarkers were assayed in cfDNA samples by qPCR. Comparison of biomarkers distribution in cases and controls was performed by a logistic regression model in both univariate and multivariate analysis. The predictive capability of each logistic model was investigated by means of the area under the ROC curve (AUC). To aid the reader to interpret the value of the AUC, values between 0.6 and 0.7, between 0.71 and 0.8 and greater than 0.8 were considered as indicating a weak predictive, satisfactory and good predictive capacity, respectively. The AUC value for each biomarker (univariate logistic model) was weak/satisfactory ranging between 0.64 (*BRAF^V600E^*) to 0.85 (total cfDNA). A good overall predictive capability for the final logistic model was found with an AUC of 0.95. The highest predictive capability was given by total cfDNA (AUC:0.86) followed by integrity index 180/67 (AUC:0.90) and methylated *RASSF1A* (AUC:0.89).

An approach based on the simultaneous determination of three biomarkers (total cfDNA, integrity index 180/67 and methylated *RASSF1A*) could improve the diagnostic performance in melanoma.

## Introduction

Molecular features of solid tumours become central in tailoring targeted therapies, but the accessibility to tumour tissue may be sometimes limited due to the size of bioptic samples or the unavailability of biological material, particularly during patients' follow up. In this context cancer-derived cell-free DNA in blood (cfDNA) represents a promising biomarker for cancer diagnosis and an useful surrogate material for molecular characterization [1].

The two classes of alterations detectable in cfDNA from cancer patients include quantitative and qualitative abnormalities. Concerning the former aspect, it is now evident that cancer patients have a higher concentration of cfDNA than healthy individuals (see ref. 2 for a review). The concentration of cfDNA is influenced by tumor stage, size, location, and other factors [3]. On the other hand, increased plasma DNA level is not a specific cancer marker, as it is observed also in patients with premalignant states, inflammation or trauma [2]. Total cfDNA concentration has been proposed as a marker for early cancer detection, but the studies conducted so far showed a scarce discriminatory power between patients and controls as well as limited sensitivity and specificity, not allowing one to reach any final conclusion on the diagnostic impact of this parameter. Several studies report a prognostic value of total cfDNA, while conflicting results have been obtained in testing this marker for therapy monitoring [3].

The reduced specificity of this quantitative test leads us to evaluate additional biomarkers reflecting qualitative alterations in cfDNA. A higher specificity in cancer diagnosis can be achieved by detecting tumor specific alterations in cfDNA, such as DNA integrity, genetic and epigenetic modifications [3]. Blood cfDNA in cancer patients originates from apoptotic or necrotic cells. In solid cancers, necrosis generates a spectrum of DNA fragments with variable size, due to random digestion by DNases. In contrast, cell death in normal blood nucleated cells occurs mostly via apoptosis that generates small and uniform DNA fragments. It has generally been observed that in patients affected by several neoplastic diseases plasma DNA contains longer fragments than in healthy subjects [4]–[10] reflected by the increase of DNA integrity index.

The above mentioned parameters can obviously be considered as non-specific biomarkers, since the increase of cfDNA concentration and integrity is common to the large majority of human solid cancers. When cfDNA is used to detect genetic and epigenetic modifications in a specific tumor, it is necessary to select definite molecular targets that are expected to be altered in affected patients. In cutaneous melanoma, the oncogene *BRAF* is frequently mutated. *BRAF* is a serine–threonine protein kinase involved in the RAS–RAF–MEK–ERK pathway [11] which regulates cell growth, survival, differentiation and senescence [12]. The oncogene *BRAF* is frequently mutated in other human cancers constitutively activating the MAPK pathway. The most common *BRAF* mutation, which accounts for more than 90% of cases of cancer involving this gene, is the T1799A transversion, converting valine to glutamic acid at position 600 (V600E) [13]. *BRAF* somatic mutations have been reported in 66% of malignant melanomas [13] and are likely to be a crucial step in the initiation of melanocytic neoplasia, as they are found also in melanocytic nevi [14]. *BRAF* mutations are an attractive target for therapeutic interventions, as they represent an early event in melanoma pathogenesis and are preserved throughout tumor progression [15]. Specific inhibitors of mutant *BRAF*, such as PLX4032, were developed and tested in clinical trials showing response rates of more than 50% and improved rates of overall and progression-free survival in patients with metastatic melanoma with the *BRAF^V600E^* genetic variant [16]. *BRAF^V600E^* mutation has been investigated as a marker in cfDNA from melanoma patients by Daniotti et al. [17] and Yancovitz et al. [18].

Finally, it is widely demonstrated that a limited number of genes is epigenetically disregulated in cutaneous melanoma. *RASSF1A* (Ras association domain family 1 isoform A) is a tumor suppressor gene, which regulates mitosis, cell cycle and apoptosis [19]. It is inactivated mostly by inappropriate promoter methylation in many types of cancers [19]. *RASSF1A* promoter is methylated in 55% of cutaneous melanomas [20]. Methylation of *RASSF1A* increases significantly with advanced clinical stage, suggesting that inactivation of this gene is associated with tumor progression [21]. *RASSF1A* promoter hypermethylation has been detected in cfDNA from melanoma patients [22]–[23] in association with a worse response to therapy and reduced overall survival [24]–[25].

Previous studies [3] assessed the diagnostic performance of each of the above mentioned biomarkers singularly considered in selected case-control comparative surveys. The aim of the present study was to identify a sequential multi-marker panel in cfDNA able to increase the predictive capability in the diagnosis of cutaneous melanoma in comparison with each single marker alone. To this purpose, we tested total cfDNA concentration, cfDNA integrity, *BRAF^V600E^* mutation and *RASSF1A* promoter methylation associated to cfDNA in a series of 76 melanoma patients and 63 healthy controls.

## Materials and Methods

### Patients and samples

Seventy six patients (32 females and 44 males, median age 63, range 23–94 years) affected by cutaneous melanoma were enrolled at the Department of Dermatological Sciences of the University of Florence. The series included: 12 patients with in situ melanoma (4 females and 8 males; age range:39–80 years, median 60 years), 49 patients with local disease (22 females and 27 males; age range:23–88 years, median 60.9 years), 5 patients with regional metastatic disease (1 females and 4 males; age range:53–88 years, median 69.4 years) and 10 patients with distant metastatic disease (5 females and 5 males; age range: 28–94 years, median 50 years). For additional baseline and clinical characteristics of invasive melanomas see Table 1.

**Table 1:** Clinicopathological characteristics of melanoma cases.

Parameter	Number of cases	Percent of cases
**Total**	76	100%
**Location**		
Head and neck	7	9.2%
Limbs	25	32.9%
Chest	40	52.7%
Acral	3	3.9%
Genital	1	1.3%
**Thickness**		
In situ	12	15.8%
≤1 mm	33	43.4%
1.01–2.0 mm	12	15.8%
2.01–4.0 mm	8	10.5%
>4 mm	11	14.5%
**Clark Level**		
I	12	15.8%
II	11	14.5%
III	19	25%
IV	34	44.7%
**Ulceration**		
Absent	58	76.3%
Present	18	23.7%
**Sentinel Lymph node**		
positive	1	1.3%
negative	20	26.3%
not done	55	72.4%
**Stage of disease**		
0	12	15.8%
IA	26	34.2%
IB	16	21.0%
IIA	7	9.2%
III	5	6.6%
IV	10	13.2%
**TNM**		
TisN0MO	12	15.8%
T1aN0M0	26	34.2%
T1bN0M0	7	9.2%
T2aN0M0	9	11.8%
T2bN0M0	3	4%
T3aN0M0	4	5.3%
T3aN1M0	2	2.6%
T3aN0M1	1	1.3%
T3bN2M1	1	1.3%
T4N1M0	3	4%
T4bN1M1	8	10.5%

As a control group 63 healthy subjects with less than 50 melanocytic nevi (median age 62, range 25–79 years) were enrolled in the study upon a dermatological examination to exclude the presence of melanoma and to provide the number of nevi. Blood samples (5 ml) were collected in EDTA tubes during the dermatologic examination and before surgery.

The research protocol was approved by the review board of the University of Florence and all the patients signed an informed consent.

Plasma was separated from blood in EDTA tubes, within three hours from blood draw by two centrifugation steps at 4°C for 10 min: at 1600 rcf and 14000 rcf, respectively. Plasma aliquots (505 µl) were stored at −80°C. DNA was extracted from 500 µl of plasma within 3 months from collection, by the QIAamp DSP Virus Kit (Qiagen, Italy) according to the manufacturer's instructions. RNAse digestion was included in the procedure to prevent RNA interference during the subsequent qPCR reactions.

### Molecular biomarkers in cfDNA

All the cfDNA samples from melanoma patients and healthy controls were submitted in duplicate to the four qPCR assays targeting the chosen biomarkers, for a total of about 1000 determinations. All the qPCR reactions were performed using the 7900HT Fast Real-Time PCR instrument (Applied Biosystems).

All the methods described in the following section have been previously developed or optimized for cfDNA by our laboratory using plasma samples from different case studies.

The total amount of cfDNA as well as the DNA integrity index were determined by two qPCR assays targeting respectively a 67 bp and a 180 bp sequence on the single copy gene *APP* (Amyloid Precursor protein, chr. 21q21.2, accession NM_000484), as already reported [26]. The primers and the hydrolysis probe for the 67 bp amplicon were previously reported [27], while for the 180 bp amplicon a different reverse primer was designed on the same target sequence [26]. The shorter amplicon (67 bp) was used to quantify total cfDNA, while the ratio between the absolute concentration of the longer amplicon (180 bp) and the shorter one (67 bp) defined the integrity index 180/67, which was used to assess the fragmentation of cfDNA. An integrity index close to 1 indicates that all the cfDNA molecules are at least 180 bp in length in the *APP* gene. An integrity index of less than 1 means that cfDNA contains fragments below 180 bp in the same target sequence. CfDNA that is more intact will be closer to a value of 1 for the integrity index.

The reactions were carried out in a 12.5 µl mix containing 1× Quantitect® Probe PCR Master Mix (QIAgen), 300 nM primers, 200 nM probe and 1 µl sample. The thermal profile of the amplification was the following: 95°C for 10 min and 45 cycles of PCR at 95°C for 15 s, 60°C for 60 s. For cfDNA quantification we used an external reference curve ranging from 10 to 10^5^ pg/tube, obtained by serial dilutions of genomic DNA extracted from a blood pool of healthy donors and measured spectrophotometrically (Nanodrop ND1000, Nanodrop, USA).

Circulating cell-free DNA bearing the mutation *BRAF^V600E^* was quantified by an allele-specific qPCR assay, as already reported [28]. The specificity for the mutated allele was conferred by the forward primer and the LNA probe. cfDNA (0.5 ng) was amplified in a reaction mixture containing 1× Quantitect® Probe PCR Master Mix (QIAgen), 200 nM primers and 200 nM probe in a final volume of 20 µl. The thermal profile of the reaction included a denaturation step at 95°C for 10 min and 50 cycles of PCR at 95°C for 15 s, 64°C for 60 s. *BRAF^V600E^* percentage was calculated by referring to a standard curve obtained by mixing DNA from mutant (SKMEL28) and wild type (MCF7) cell lines in the following proportions: 100%, 50%, 20%, 10%, and 1% mutated alleles. The presence of the *BRAF^V600E^* mutation was excluded in the MCF7 human breast adenocarcinoma cell line and confirmed in the SKMEL28 human melanoma cell line by High Resolution melting followed by sequencing (data not shown).

Subsequently *BRAF^V600E^* concentration was expressed in nanograms per ml plasma by multipling this percentage for absolute DNA concentration determined by the qPCR assay for *APP*.

The methylated form of *RASSF1A* promoter was quantified in plasma after digesting unmethylated DNA by a methylation-sensitive enzyme: 5 µl of plasma DNA were treated with 10 units of Bsh1236I (Fermentas, Canada) in a reaction volume of 25 µl at 37°C for 16 hours. Subsequently, 5 µl of enzyme-treated DNA underwent a qPCR assay for *RASSF1A* promoter, in a final volume of 25 µl, according to the protocol already described by Chan et al. [29]. A reference curve obtained by serial dilutions of genomic DNA was used to quantify the methylated alleles. Results were expressed as genomic equivalents (GE, each corresponding to 6.6 pg DNA) per ml plasma.

### Statistical Analysis

All the considered biomarkers were analysed as continuous variables in their original scale or after an appropriate transformation. Comparison of biomarkers distribution in cases and controls overall as well as according to stage of disease was performed by using the Kolmogorov-Smirnov test [30]. The relationship between each biomarker and the disease status was investigated by resorting to a logistic regression model in both univariate and multivariate fashion [31]. In the logistic regression model, each regression coefficient is the logarithm of the odds ratio (OR). Under the null hypothesis of no association, the value of OR is expected to be 1.00. The hypothesis of OR = 1 was tested using the Wald Statistic. For each model the biomarker that was statistically significant (alpha = 0.05) in univariate analysis was considered in the initial model of multivariate analysis. A final more parsimonious model was then obtained using a backward selection procedure in which only the variables reaching the conventional significance level of 0.05 were retained (final model). The relationship between each biomarker and disease status was investigated by resorting to a regression model based on restricted cubic splines. The most complex model considered was a four-nodes cubic spline with nodes located at the quartiles of the distribution of the considered biomarker [32]. The contribution of non-linear terms was evaluated by the likelihood ratio test.

We investigated also the predictive capability (ie diagnostic performance) of each logistic model by means of the area under the ROC curve (AUC) [33].

This curve measures the accuracy of biomarkers when their expression is detected on a continuous scale, displaying the relationship between sensitivity (true-positive rate, y-axes) and 1-specificity (false-positive rate, x-axes) across all possible threshold values of the considered biomarker. A useful way to summarize the overall diagnostic accuracy of the biomarker is the area under the ROC curve (AUC) the value of which is expected to be 0.5 in absence of predictive capability, whereas it tends to be 1.00 in the case of high predictive capacity [33]. To aid the reader to interpret the value of this statistic, we suggest that values between 0.6 and 0.7 be considered as indicating a weak predictive capacity, values between 0.71 and 0.8 a satisfactory predictive capacity and values greater than 0.8 a good predictive capacity [34].

Finally the contribution of each variables to the predictive capability of the final model was investigated by comparing the AUC value in the model with that of the same model without the variable itself. All statistical analyses were performed with the SAS software (Version 9.2.; SAS Institute Inc. Cary, NC) by adopting a significance level of alpha = 0.05.

## Results

**Figure 1:**
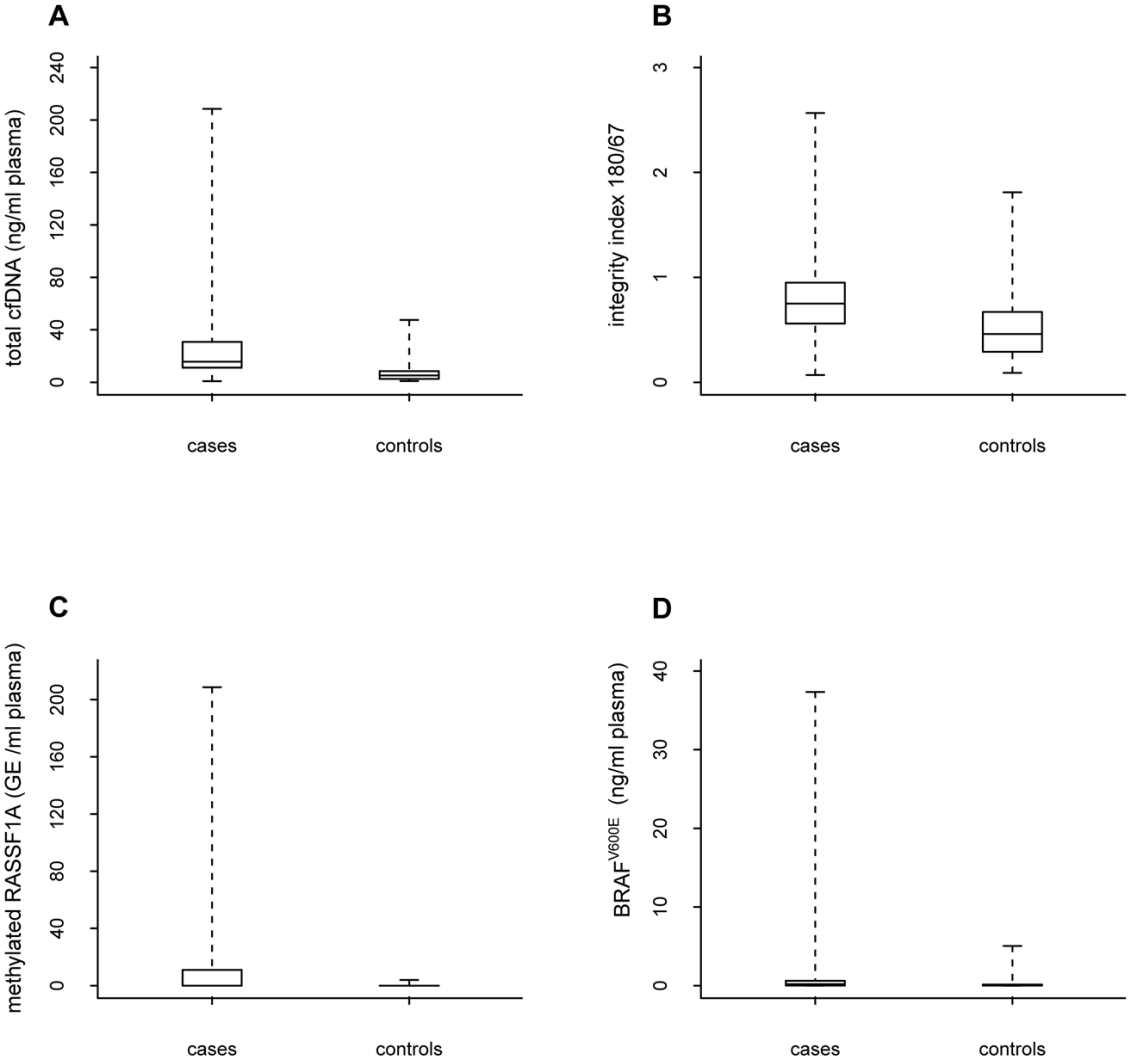
Biomarkers distribution in cases and controls. Box plots reflecting the distribution in cases and controls of total cfDNA (Panel A), integrity index 180/67 (Panel B), methylated *RASSF1A* (Panel C), and *BRAF^V600E^* (Panel D). Each box indicates the 25^th^ and 75^th^ percentiles. The horizontal line inside the box indicates the median, and the whiskers indicate the extreme measured values.

The box-plots reported in Figure 1, panel A–D, describe the distribution of each biomarker in case and controls.

Table **2** reports some descriptive statistics of these distributions. Using the Kolmogorov–Smirnov test, we found that the difference of the distributions of each biomarker in cases and controls was statistically significant (p-value <0.05). As reported in supplemental Table S1, the same results were observed when this comparison was performed according to the stage of disease for cfDNA and integrity index 180/67. Conversely these findings were not observed within stage I–II for methylated *RASSF1A* and within stage 0 and stage III–IV for *BRAF^V600E^*.

**Table 2:** Descriptive Statistics.

biomarker		min	25^th^ centile	median	75^th^ centile	max	IQR	p-value†
total cfDNA (ng/ml plasma)	cases	0.894	11.098	15.641	30.785	208.560	19.687	<0.0001
	controls	0.990	2.530	5.260	8.740	47.490	6.210	
integrity index 180/67	cases	0.070	0.560	0.750	0.950	2.568	0.390	<0.0001
	controls	0.090	0.290	0.460	0.670	1.810	0.380	
methylated *RASSF1A* (GE/ml plasma)	cases	0.000	0.000	0.000	11.040	208.680	11.040	0.0003
	controls	0.000	0.000	0.000	0.000	4.010	0.000	
*BRAF^V600E^* (ng/ml plasma)	cases	0.000	0.006	0.200	0.610	37.338	0.603	0.0012
	controls	0.000	0.010	0.080	0.163	5.060	0.153	

Abbreviations: IQR, Interquartile range (75th centile – 25th centile).

† p-value of the Kolmogorov-Smirnov test by comparing the distribution of cases and controls.

For all the biomarkers considered in the logistic regression model we found that a linear relationship between the log odds and their values on the original (methylated *RASSF1A*) or logarithm (total cfDNA, integrity index 180/67 and *BRAF^V600E^*) scale was appropriate. As reported in Table 3, disease status was significantly associated with all the biomarkers in the logistic univariate analysis. Consequently the initial model of the logistic multivariate regression analysis was built by including all four biomarkers. As reported in Table 4, total cfDNA, integrity index 180/67 and methylated *RASSF1A* retained a statistically significant (p-value <0.05) association with disease status in the multivariate final logistic model.

**Table 3:** Univariate logistic analysis.

biomarker	OR^a^	OR 95%CI	p-value†	AUC	AUC 95%CI	p-value
total cfDNA (ng/ml plasma)	5.621	3.102–10.185	<0.0001	0.853	0.788–0.918	<0.0001
integrity index 180/67	4.790	2.356–9.740	<0.0001	0.759	0.677 −0.840	<0.0001
methylated *RASSF1A* (GE/ml plasma)	1.413	1.112–1.795	0.005	0.688	0.621 −0.754	<0.0001
*BRAF^V600E^* (ng/ml plasma)	6.061	1.650–22.263	0.007	0.635	0.540–0.730	0.005

Abbreviations: OR, Odds Ratio; CI, Confidence Interval; AUC, area under the ROC curve.

a Odds Ratio for any increase of one unit.

† p-value of the Wald statistic.

**Table 4:** Final multivariate logistic model.

biomarker	OR^a^	OR 95%CI	p-value†	AUC	AUC 95%CI	p-value
total cfDNA (ng/ml plasma)	6.592	3.084–14.088	<0.0001	0.945	0.910–0.980	<0.0001
integrity index 180/67	7.783	2.944–20.579	<0.0001			
methylated *RASSF1A* (GE/ml plasma)	1.450	1.100–1.910	0.008			

Abbreviations: OR, Odds Ratio; CI, Confidence Interval; AUC, area under the ROC curve.

a Odds Ratio for any increase of one unit.

† p-value of the Wald statistic.

**Figure 2:**
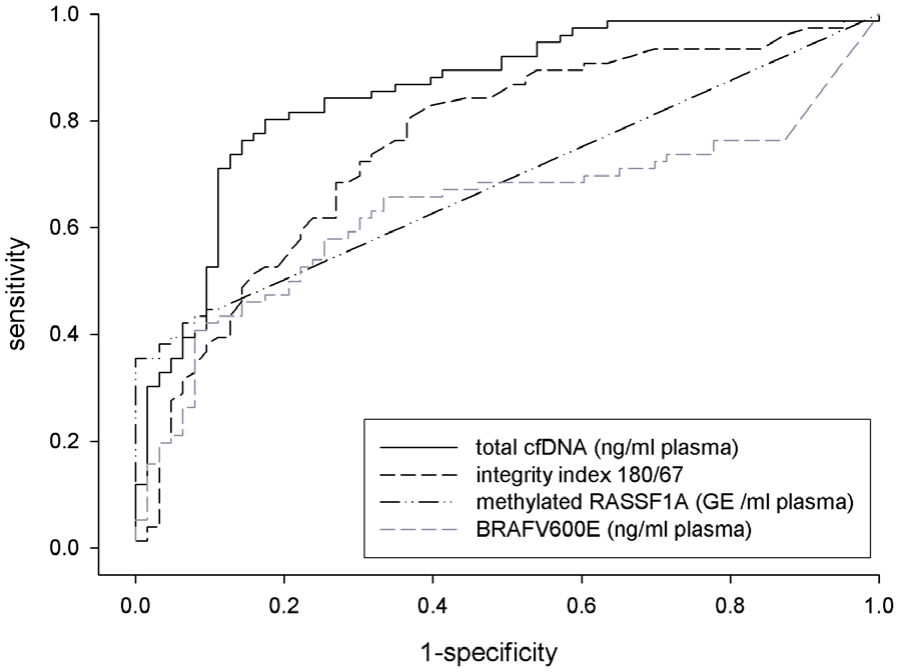
ROC Curves deriving from the univariate logistic analysis. ROC curves derived from the univariate logistic analysis corresponding to total cfDNA (AUC = 0.85), integrity index 180/67 (AUC = 0.76), methylated *RASSF1A* (AUC = 0.69) and *BRAF^V600E^* (AUC = 0.64).

The AUC values computed for each biomarker (univariate logistic model) indicated a weak/satisfactory level of predictive capability by ranging between 0.64 (*BRAF^V600E^*) to 0.85 (total cfDNA) (Table 3 and Figure 2). Of note for all the considered biomarkers the 95% Confidence Interval (95%CI) of the AUC fails to include the 0.5 value (i.e. absence of predictive capability).

**Figure 3:**
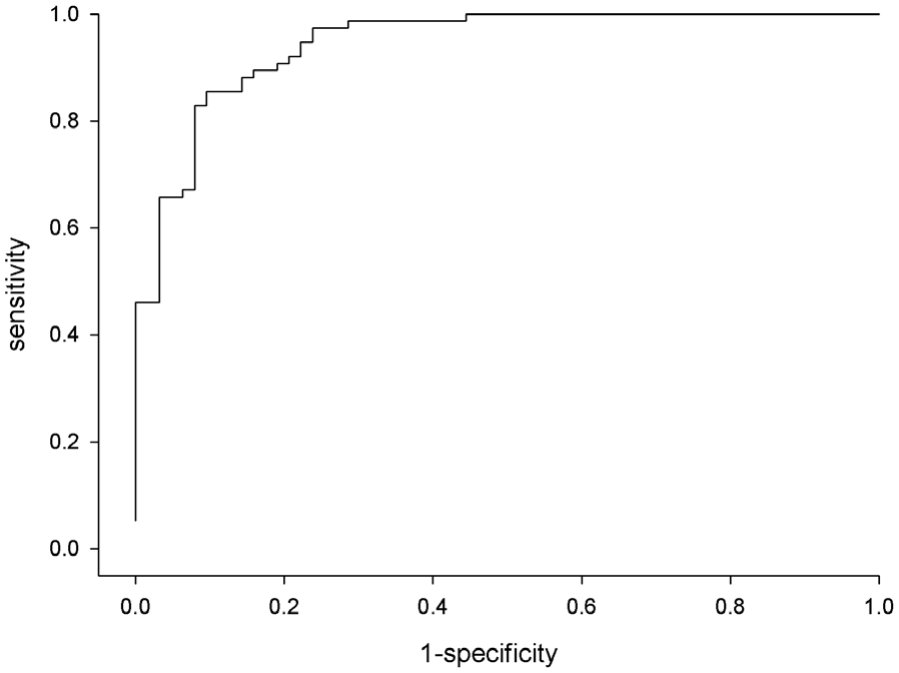
ROC Curve deriving from the multivariate final logistic model. ROC curve derived from the final multivariate logistic model (AUC = 0.95).

**Figure 4:**
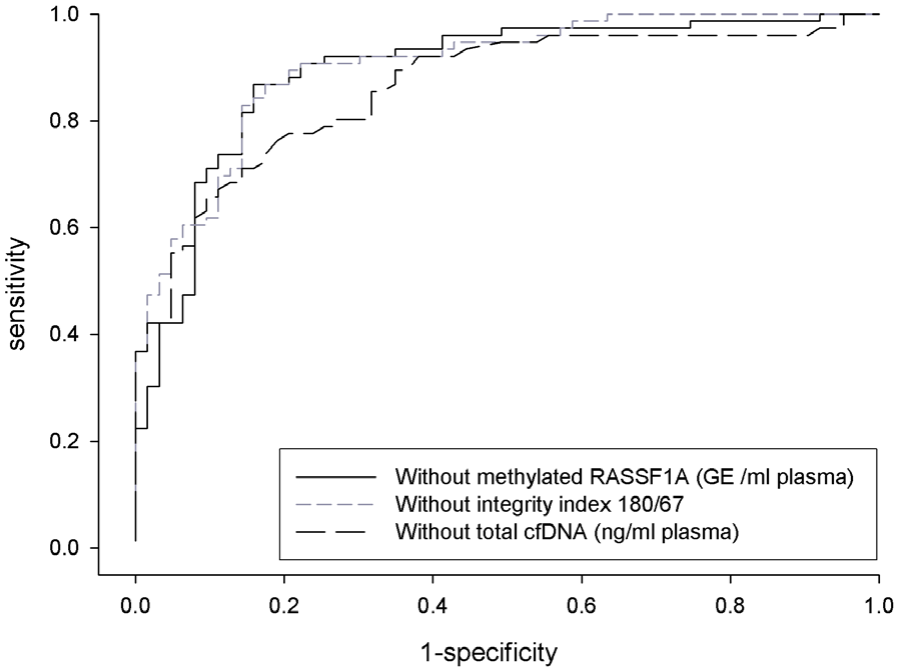
Contribution of each biomarker to the final model - ROC Curves. ROC curves corresponding to the contribution of each biomarker in the final multivariate logistic model. Without total cfDNA (AUC = 0.86), without integrity index 180/67 (AUC = 0.90), without methylated *RASSF1A* (AUC = 0.89).

Overall, a good predictive capability was observed for the final logistic model with an AUC of 0.95 (95% CI: 0.91–0.98) (Table 4 and Figure 3). The contribution of each variable of the final model to the diagnostic performance is shown in Table 5 and graphically described in Figure 4. The highest predictive capability was given by total cfDNA (AUC:0.86, 95%CI: 0.80–0.92) followed by integrity index 180/67 (AUC:0.90, 95%CI: 0.85–0.95) and methylated *RASSF1A* (AUC:0.89, 95%CI: 0.84–0.95). As shown in the supplemental figure S1 a comparable predictive capability was observed for each considered biomarker (univariate analysis) according to the stage of disease. Only for BRAF^V600E^ within the stage 0 and stage III–IV the 95% CI of the AUC includes the 0.5 value.

**Table 5:** Contribution of each biomarker of the final model.

	AUC	AUC 95%CI	p-value
Final model	0.945	0.910–0.980	<0.0001
Without the following variables:			
total cfDNA (ng/ml plasma)	0.862	0.801–0.923	<0.0001
integrity index 180/67	0.903	0.854–0.952	<0.0001
methylated RASSF1A (GE/ml plasma)	0.894	0.839–0.950	<0.0001

Abbreviations: AUC, area under the ROC curve; CI, Confidence Interval.

## Discussion

The analysis of cfDNA may have the potential to complement or replace the existing cancer tissue and blood biomarkers in the future [35]. In order to reach this goal, specific and sensitive analytical procedures must be developed and optimized to compute proper circulating target molecules showing differences between patients and healthy subjects. It is now widely accepted that a single biomarker cannot fully distinguish between controls and patients and consequently an approach based on different markers would be preferable in order to achieve a stronger predictive ability [36].

It has been demonstrated that in prenatal screening, a combination of multiple markers, each with limited sensitivity and/or specificity, can lead to a more powerful screening test [37]. Similarly, Schneider and Mizejewski [38] suggest to develop a multi-marker screening approach for cancer diagnosis. Unfortunately this strategy has been proven unsuccessful, notwithstanding the high number of new biomarkers reported in the literature, even if some examples on prostate ovarian and colorectal cancer clearly showed that multi-marker screening can have its place in early cancer detection [38]–[39].

The study presented here tests the diagnostic potential of four markers associated to cfDNA in identifying melanoma patients. Particular efforts were dedicated to the technical aspects of the methods adopted for each single parameter allowing to reach accurate and reproducible measurements. We evaluated total cfDNA concentration by a qPCR assay for the single copy gene *APP*, as well as DNA fragmentation represented by the integrity index 180 bp/67 bp (see Materials & Methods). On the other hand, tumour contribution to cfDNA was assessed by quantifying *BRAF^V600E^* mutated alleles and *RASSF1A* promoter methylation. These markers have been used in a panel in all patients, thus representing a simple model potentially adoptable by any laboratory. Following the standard approach for the clinical validation of biomarkers for early detection [40] the next step will be focused on the assessment of the impact of these biomarkers on clinical practice including the identification of the most suitable thresholds to use for the early detection of melanoma by clinicians.

**Figure 5:**
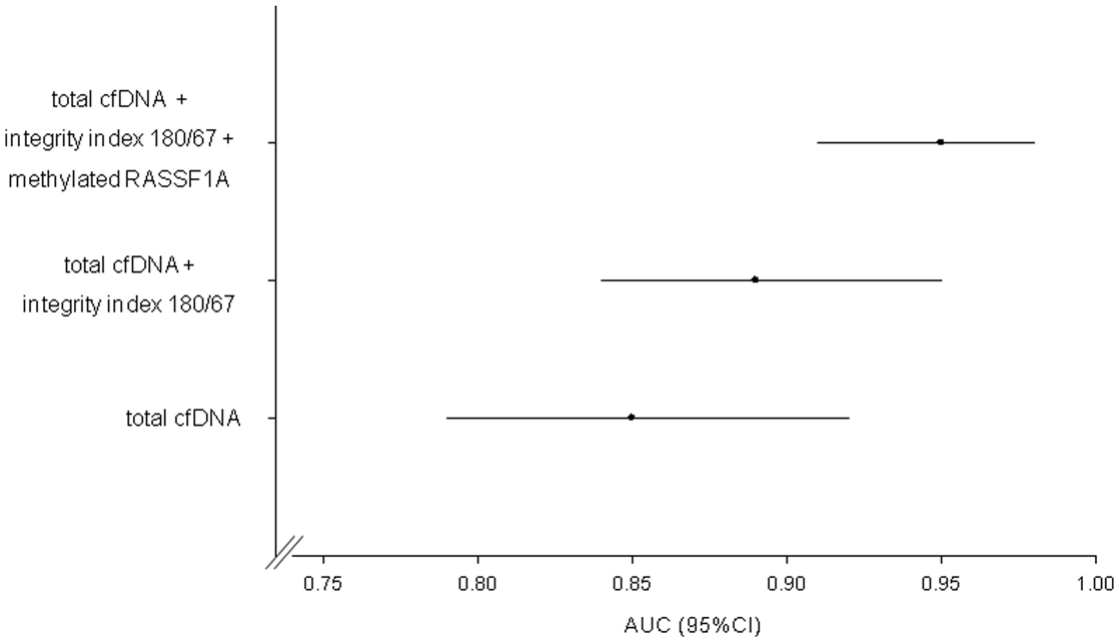
Sequential approach. Diagnostic performance increment (in terms of AUC) achieved by moving from cfDNA alone (AUC = 0.85; 95%CI = 0.79–0.92) to cfDNA and integrity index 180/67 (AUC = 0.89; 95%CI = 0.84–0.95) and to cfDNA, integrity index 180/67 and methylated *RASSF1A* (AUC = 0.95; 95%CI = 0.91–0.98).

Our preliminary results show that by jointly considering the panel of biomarkers here investigated the highest predictive capability is given by total cfDNA followed by integrity index 180/67 and methylated *RASSF1A*. According to these results, an approach based on the simultaneous determination of the three biomarkers (total cfDNA, integrity index 180/67 and methylated *RASSF1A*) could be suggested to improve the diagnostic performance in melanoma. Alternatively, as reported in Figure 5, a more parsimonious sequential approach could be adopted using pre-selection by cfDNA, followed by further selection using integrity index 180/67 and/or methylated *RASSF1A*.

We plan to evaluate the prognostic role of both these approaches as soon as the follow-up time of our case study will be adequate (5 years). However preliminary data (not shown), obtained in a subgroup of patients submitted to an additional blood draw 2 weeks after surgery, show a decrease of the four biomarkers, suggesting the potential role of these test as useful tools for monitoring patients after initial diagnosis/surgery.

Even though each biomarker investigated in the present work is not exclusively associated with melanoma, their combination reveals a high specificity for melanoma detection.

## Supporting Information

Figure S1 **95%CI of the AUC according to the stage of disease.** Bonferroni adjusted confidence intervals of the AUC of total cfDNA (Panel A), integrity index 180/67 (Panel B), methylated RASSF1A (Panel C), and BRAFV600E (Panel D) according to the stage of disease. The horizontal dashed line in each Panel represent the AUC value obtained for each biomarker by comparing all cases and controls.
(TIF)

Table S1 **Descriptive Statistics according to the stage of disease.**
(DOC)
