# Supplementary material for: Multiparametric Analysis of Cell-Free DNA in Melanoma Patients
Source: PLoS One. 2012 Nov 27;7(11):e49843. doi: 10.1371/journal.pone.0049843 (PMC3507877; doi:10.1371/journal.pone.0049843)
Supplement: Table S1 — Descriptive Statistics according to the stage of disease. (DOC) [file pone.0049843.s002.doc]

**Table S1**. Descriptive Statistics according to the stage of disease

| **biomarker** | **Stage of disease** | **n** | **min** | **25^th^ centile** | **median** | **75^th^ centile** | **max** | **IQR** | **p-value†*** |
| --- | --- | --- | --- | --- | --- | --- | --- | --- | --- |
| total cfDNA  (ng/ml plasma) | 0 | 12 | 5.824 | 11.644 | 19.481 | 32.970 | 48.370 | 21.326 | <0.001 |
|  | I-II | 49 | 3.528 | 11.322 | 15.390 | 27.300 | 208.560 | 15.978 | <0.001 |
|  | III-IV | 15 | 0.894 | 7.211 | 17.701 | 32.090 | 125.510 | 24.880 | 0.003 |
|  | controls ^a^ | 63 | 0.990 | 2.530 | 5.260 | 8.740 | 47.490 | 6.210 | - |
| integrity index 180/67 | 0 | 12 | 0.460 | 0.650 | 0.745 | 0.910 | 1.393 | 0.260 | 0.010 |
|  | I-II | 49 | 0.070 | 0.560 | 0.750 | 0.960 | 2.568 | 0.400 | <0.001 |
|  | III-IV | 15 | 0.330 | 0.530 | 0.820 | 0.950 | 1.270 | 0.420 | 0.020 |
|  | controls ^a^ | 63 | 0.090 | 0.290 | 0.460 | 0.670 | 1.810 | 0.380 | - |
| methylated *RASSF1A*  (GE /ml plasma) | 0 | 12 | 0.000 | 0.000 | 4.840 | 9.545 | 21.060 | 9.545 | 0.018 |
|  | I-II | 49 | 0.000 | 0.000 | 0.000 | 3.520 | 71.400 | 3.520 | 0.183 |
|  | III-IV | 15 | 0.000 | 0.000 | 15.650 | 36.150 | 208.680 | 36.150 | <0.001 |
|  | controls ^a^ | 63 | 0.000 | 0.000 | 0.000 | 0.000 | 4.010 | 0.000 | - |
| *BRAF^V600E^*  (ng/ml plasma) | 0 | 12 | 0.000 | 0.003 | 0.172 | 0.790 | 4.903 | 0.788 | 0.673 |
|  | I-II | 49 | 0.000 | 0.090 | 0.266 | 0.566 | 6.808 | 0.476 | 0.001 |
|  | III-IV | 15 | 0.000 | 0.000 | 0.038 | 0.960 | 37.338 | 0.960 | 0.981 |
|  | controls ^a^ | 63 | 0.000 | 0.010 | 0.080 | 0.163 | 5.060 | 0.153 | - |

Abbreviations: IQR, Interquartile range (75th centile – 25th centile).

† p-value of the Kolmogorov-Smirnov test by comparing the distribution in cases and control according to the each stage of disease.

* Bonferroni adjustment

^a^ References category
